# Cerebrospinal fluid ctDNA as a diagnostic and prognostic tool in gliomas: a systematic review and meta-analysis

**DOI:** 10.3389/fonc.2025.1714287

**Published:** 2025-12-11

**Authors:** Rebeca Pérez-Alfayate, Mateo Paz-Cabezas, Pedro Pérez-Segura, Rafael Sanchez del Hoyo, Santiago Cabezas-Camarero

**Affiliations:** 1 Neurosurgery Department, Hospital Clínico Universitario San Carlos, Instituto de Investigación Sanitaria del Hospital Clínico San Carlos (IdISSC), Madrid, Spain; 2 Clinical and Translational Research in Oncology group, Molecular Oncology Laboratory, Hospital Clínico Universitario San Carlos, Instituto de Investigación Sanitaria del Hospital Clínico San Carlos (IdISSC), Madrid, Spain; 3 Medical Oncology Department, Hospital Clínico Universitario San Carlos, Instituto de Investigación Sanitaria del Hospital Clínico San Carlos (IdISSC), Madrid, Spain; 4 Research Methodological Support Unit and Preventive Department, Hospital Clínico San Carlos, Instituto de Investigación Sanitaria del Hospital Clínico San Carlos (IdISSC), Madrid, Spain

**Keywords:** glioma, cerebrospinal fluid, circulating tumor DNA (ctDNA), liquid biopsy, meta-analysis

## Abstract

**Background:**

Liquid biopsy using circulating tumor DNA (ctDNA) has emerged as a promising tool for molecular characterization and monitoring in gliomas. This systematic review and meta-analysis evaluated the diagnostic and prognostic value of ctDNA in cerebrospinal fluid (CSF), compared to plasma, as well as factors influencing its detection.

**Methods:**

We systematically reviewed studies published between 2015 and 2025 reporting on ctDNA detection in CSF from adult glioma patients. Pooled analyses compared detection rates between CSF and plasma, CSF collection routes, assay types (targeted vs. bespoke), and IDH mutation status. Molecular concordance with tumor tissue and clinical correlations were also assessed.

**Results:**

Twelve studies comprising 388 patients with WHO grade II–IV gliomas were included. ctDNA detection in CSF was achieved in 82% of patients, compared with only 16% in plasma. Tumor–CSF molecular concordance was 90% (95% CI 86–93). Detection was significantly higher in CSF than in plasma (OR 0.05, 95% CI 0.01–0.24). No significant differences were observed between IDH-wildtype and IDH-mutant gliomas (OR 0.72, 95% CI 0.26–2.02) or between intracranial and lumbar CSF collection techniques (p > 0.9).

**Conclusions:**

CSF outperforms plasma for ctDNA-based molecular profiling in gliomas, offering both diagnostic and prognostic applications. Detection is numerically higher in IDH-wildtype gliomas, underscoring its potential role as a biomarker in this subgroup. While no significant differences were observed between collection routes in the pooled analysis, single-study evidence suggests a possible advantage of intracranial sampling, which requires further prospective evaluation. Its integration into clinical workflows may aid in cases where tissue biopsy is not feasible. Standardized methodologies and prospective multicenter validation are needed to enable routine clinical implementation.

## Introduction

Gliomas are the most common primary malignant brain tumors in adults, with glioblastoma (GBM) being the most aggressive subtype and associated with a poor prognosis despite surgery, radiotherapy, and chemotherapy. Molecular profiling has become essential for accurate diagnosis, classification, and therapeutic decision-making, particularly following the 2021 WHO classification update (1).

Traditionally, molecular characterization relies on tissue biopsies. However, this approach presents several limitations. Surgical access to deep-seated or eloquent brain regions may be contraindicated or high-risk, and even when feasible, sampling may yield insufficient or non-representative material (2). Moreover, the intrinsic spatial heterogeneity of gliomas means that a single biopsy may not fully capture the tumor’s molecular landscape. This can lead to underrepresentation of critical subclonal alterations that may have diagnostic or therapeutic significance (3).

In this context, liquid biopsy has emerged as a promising and minimally invasive strategy to overcome some of the limitations of conventional tissue sampling. Circulating tumor DNA (ctDNA), particularly when obtained from cerebrospinal fluid (CSF), offers higher sensitivity than plasma-based assays for detecting tumor-specific alterations in brain tumors. This is likely due to the limited permeability of the blood–brain barrier, which restricts the release of tumor DNA into the systemic circulation (4). CSF-based ctDNA analysis has shown potential in identifying key mutations such as IDH1 or IDH2, TERT promoter variants, and EGFR alterations, using platforms including digital PCR and next-generation sequencing (5). Nevertheless, substantial heterogeneity exists across published studies. Variations in analytical platforms, sequencing coverage, tumor subtypes included, and clinical timing of sampling all contribute to inconsistent results. Additionally, preanalytical factors such as the method of CSF collection, whether by lumbar puncture or alternative techniques such as subarachnoid, intracisternal, or intraventricular access, may influence the concentration of ctDNA recovered and affect detection sensitivity (6).

To address these gaps, we conducted a systematic review and meta-analysis of ctDNA detection in glioma patients, focusing on studies analyzing CSF and plasma samples. Our primary objectives were to compare detection rates across four clinically and methodologically relevant variables: biospecimen type, specifically CSF versus blood; CSF collection route, comparing lumbar puncture to cranial approaches such as subarachnoid, cisternal, or intraventricular sampling; the type of molecular assay, distinguishing targeted panels aimed at known alterations from broader, bespoke approaches, including differences in sequencing platforms such as next-generation sequencing or digital PCR; and IDH mutation status, comparing IDH-wildtype versus IDH-mutant gliomas.

This work aims to clarify the current evidence, identify methodological limitations, and support the development of more standardized and clinically useful liquid biopsy strategies in glioma.

## Methods

### Study design and objectives

This systematic review and meta-analysis aimed to evaluate the diagnostic and prognostic utility of circulating tumor DNA (ctDNA) in the cerebrospinal fluid (CSF) of patients with histologically confirmed gliomas. Specifically, we assessed whether CSF-derived ctDNA reliably reflects the molecular profile of the primary tumor (diagnostic value) and whether its presence correlates with clinical features such as progression-free survival (PFS) and overall survival (OS), tumor grade, or tumor burden (prognostic value). We also examined detection rates across biospecimen types (CSF vs. plasma), CSF collection routes, and molecular techniques, including targeted versus bespoke assays and different sequencing platforms. This study was conducted in accordance with the *Preferred* Reporting *Items for Systematic Reviews and Meta-Analyses* (PRISMA) 2020 guidelines.

### Search strategy

A comprehensive literature search was conducted across Pubmed and Embase databases to identify studies investigating liquid biopsy for genomic profiling in glioma patients. The search spanned from January 1, 2015 to June 30, 2025. Multiple keyword combinations were used to ensure broad coverage. The following search strategies were employed:

- (“glioma” OR “glioblastoma” OR “astrocytoma” OR “oligodendroglioma”) AND (“liquid biopsy” OR “ctDNA” OR “circulating tumor DNA”) AND (“plasma” OR “blood”) AND (“cerebrospinal fluid” OR “CSF”) AND (“mutation” OR “genomic profiling” OR “molecular analysis”)
- (“glioma” OR “glioblastoma”) AND (“cerebrospinal fluid” OR “CSF”) AND (“lumbar puncture” OR “intracranial sampling” OR “ventricular drainage” OR “cisternal puncture”) AND (“liquid biopsy” OR “ctDNA”)
- (“glioma” OR “glioblastoma”) AND (“liquid biopsy” OR “ctDNA”) AND (“targeted sequencing” OR “bespoke panel” OR “custom panel”)
- (“glioma” OR “glioblastoma”) AND (“liquid biopsy” OR “ctDNA”) AND (“next-generation sequencing” OR “NGS”) AND (“digital PCR” OR “droplet digital PCR” OR “ddPCR”)
- (“glioma” OR “glioblastoma”) AND (“liquid biopsy” OR “ctDNA”) AND (“IDH” OR “IDH1” OR “IDH2”) AND (“wild-type” OR “mutant”)

**Figure 1:**
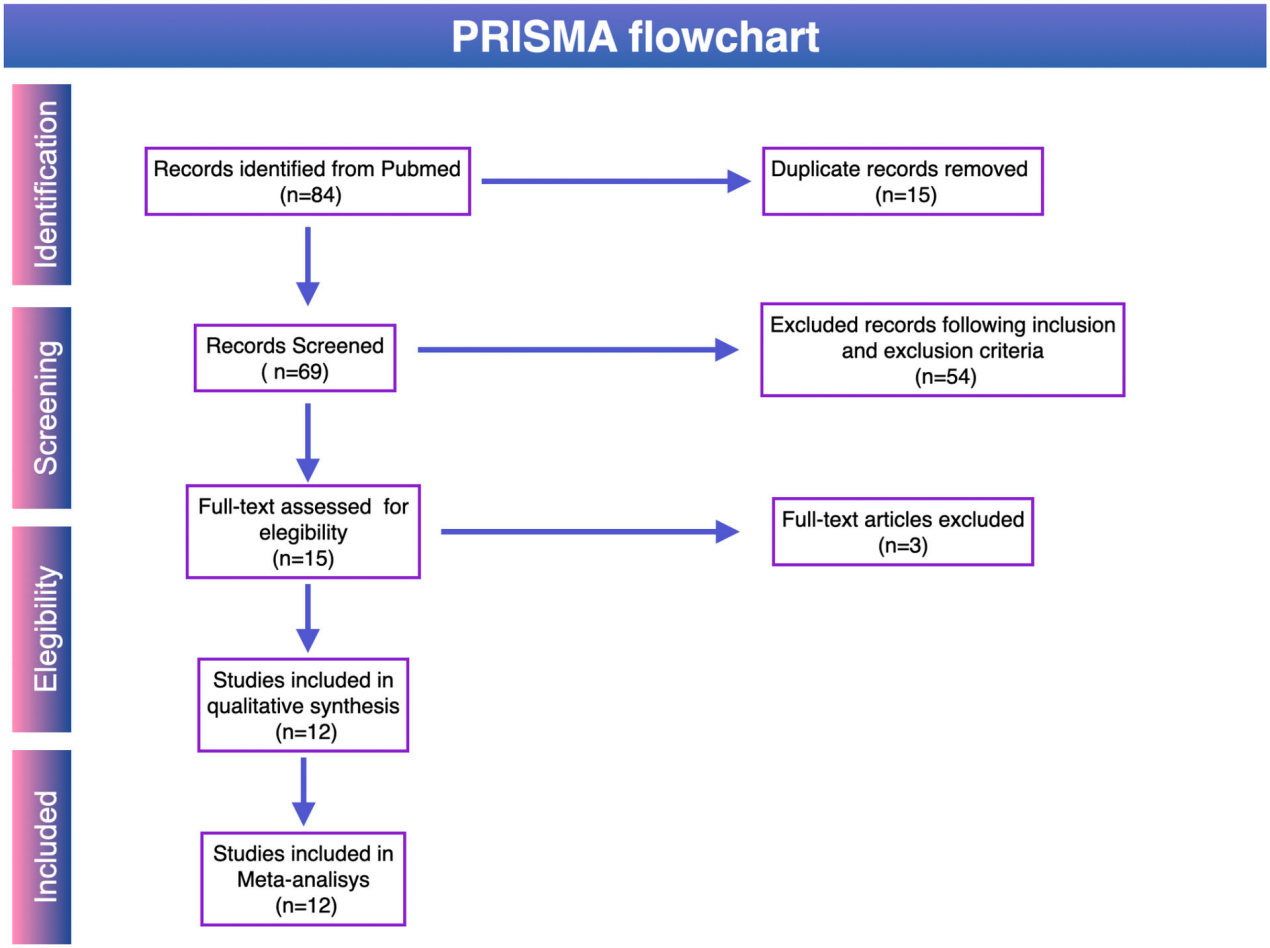
PRISMA 2020 flow diagram of study selection. Flow diagram summarizing identification, screening, and inclusion of studies. A total of 84 records were identified, 15 duplicates were removed, 69 records were screened, and 15 full-text articles were assessed. Twelve studies met eligibility criteria and were included in the qualitative and quantitative analyses.

All identified references were imported into a reference manager and de-duplicated. Two independent reviewers screened titles and abstracts, followed by full-text evaluation of potentially eligible studies. The selection process followed PRISMA 2020 guidelines and is summarized in **Figure 1**.

### Eligibility criteria

Studies were included if they met the following criteria: involved adult patients with glioma of any histologic or molecular subtype, used CSF as a biospecimen for liquid biopsy, and presented original clinical data from case series, cohort studies, or clinical trials. To be eligible, studies had to report on the diagnostic or prognostic role of CSF-derived biomarkers, including ctDNA detection rates, concordance with tumor tissue, or associations with clinical outcomes. No restrictions were applied based on whether sensitivity, specificity, area under the curve (AUC), or hazard ratios (HRs) were reported. Only studies published in English between January 1, 2015 and June 30, 2025 were considered.

Exclusion criteria included: review articles, editorials, or conference abstracts without original data; single case reports; preclinical studies based solely on cell lines or animal models; studies focusing on non-glioma brain tumors or mixed cohorts including metastases; duplicate reports or secondary analyses of previously published datasets; unclear or non-relevant liquid biopsy methodologies; studies focusing on pediatric patients (under 18 years of age); and studies with known cases of leptomeningeal carcinomatosis, given its confounding impact on ctDNA levels in CSF.

All studies meeting inclusion criteria were subjected to full-text screening for final data extraction and risk-of-bias assessment.

### Data extraction

A standardized form was used to extract data from each eligible study. Variables collected included: first author, year of publication, country, study design (prospective or retrospective), sample size, glioma classification per WHO 2021, and characteristics of the liquid biopsy approach. Technical variables included biospecimen type (CSF, plasma), ctDNA target genes, method of fluid collection (e.g., lumbar puncture, Omaya reservoir, intraoperative subarachnoid, cisternal, or intraventricular access), and detection platform (e.g., targeted next-generation sequencing, droplet digital PCR, BEAMing, whole-exome sequencing). Reported diagnostic and prognostic outcomes were also collected, including sensitivity, specificity, AUC, and HRs. When relevant outcomes were mentioned but not clearly tabulated, data were cross-checked in **Supplementary Materials** or extracted manually from the text or figures.

For subgroup analyses, ‘intracranial access’ was defined as CSF collection obtained directly from the cranial compartment, including intraoperative subarachnoid, cisternal, or ventricular sampling performed either through direct puncture or via indwelling devices such as ventricular catheters or Ommaya reservoirs. These approaches were grouped together due to their shared anatomical proximity to the tumor and ventricular system.

For the purposes of pooled analysis, the study by Orzan et al. (7) was split into two independent cohorts (intracranial vs. lumbar puncture CSF collection) to enable comparison of collection routes. In addition, in the study by Cabezas-Camarero et al. (3) a single IDH-mutant case obtained by lumbar puncture was excluded, so that the remaining cohort could be consistently integrated into the intracranial CSF group.

### Risk of bias assessment and statistical analysis

The methodological quality of included studies was assessed using the Newcastle–Ottawa Scale (NOS), suitable for observational studies. The NOS evaluates study quality across three domains: selection of participants, comparability of groups, and outcome ascertainment. Each study was scored independently by two reviewers, with a maximum score of 9 points. Studies were categorized as low risk of bias (≥7), moderate risk (5–6), or high risk (<5). Discrepancies were resolved by discussion and consensus.

### Statistical analysis

A meta-analysis of proportions was conducted using R version 4.3.2 with the *meta* and *metafor* packages. Studies reporting proportions of liquid biopsy detection in gliomas were included, and results were summarized descriptively without formal hypothesis testing (i.e., no p-values). Overall pooled proportions were estimated and displayed as forest plots with 95% confidence intervals. The choice between fixed- and random-effects models was guided by the assessment of heterogeneity, using Cochran’s Q test and the I² statistic. When heterogeneity was low and not statistically significant, fixed-effect models were applied; otherwise, random-effects models were used. Specifically, pooled estimates were obtained for IDH-mutant and IDH-wildtype detection in CSF, overall CSF detection rates, plasma detection rates, and tumor–CSF concordance. Comparative analyses using Mantel–Haenszel weighting were performed to calculate odds ratios for CSF detection in IDH-wildtype versus IDH-mutant gliomas, and for positivity in plasma versus CSF. Subgroup analyses were additionally carried out to evaluate the effect of CSF collection method (intracranial vs. lumbar puncture) on detection rates. For this subgroup analysis, a meta-regression model was fitted with the collection method included as a categorical moderator. The between-study variance component was estimated using the restricted maximum likelihood (REML) approach, and the Knapp–Hartung adjustment was applied to obtain more robust standard errors and confidence intervals for the moderator effect.

To evaluate the robustness of pooled estimates, we conducted leave-one-out (LOO) case-deletion analyses for all meta-analyses. In each iteration, one study was omitted and the model was re-estimated using the same parameters as in the primary analysis. The resulting pooled estimate, its 95% confidence interval, and heterogeneity metrics were compared with those from the full model. For sparse or zero-event data, standard continuity-correction procedures recommended for dichotomous outcomes were applied to ensure stable estimation. Robustness was judged based on the magnitude and direction of changes and the stability of statistical inference.

## Results

Following the application of PRISMA 2020 guidelines (**Figure 1**), twelve studies (3, 4, 7–16) were selected for inclusion in this meta-analysis, comprising a total of 388 adult patients with histologically confirmed gliomas. The primary reasons for exclusion of other articles were studies conducted in pediatric populations (patients <18 years) (6, 17–30) a focus on non-ctDNA biomarkers such as cell-free DNA (cfDNA) or circulating tumor cells (CTCs) (2, 31–42), the study of non-glioma or metastatic brain tumors (43) (44), evaluate of spinal pathology (45, 46), single-case clinical reports (47, 48), or being review articles and meta-analyses without original patient data (18, 49–64). All selected studies analyzed cerebrospinal fluid (CSF) for ctDNA detection, and all also included primary tumor tissue analysis, with some additionally incorporating plasma samples. Despite methodological differences in CSF sampling routes and molecular platforms, all studies shared the common objective of evaluating the diagnostic or prognostic utility of CSF-derived ctDNA in gliomas. A full list of included and excluded studies, along with reasons for exclusion, is provided in **Supplementary Material 1** (**Supplementary Tables 1**, **2**). Risk of bias assessment using the Newcastle–Ottawa Scale (NOS) showed scores ranging from 5 to 8, with 9 studies rated as low risk and 3 as moderate risk; no study was judged to be at high risk of bias (**Supplementary 1**, **Table 3**).

The twelve included cohorts encompassed WHO grades II–IV. Primary tumor tissue was analyzed in all 12 studies, CSF in 12, and plasma in 6; several studies included more than one specimen type. CSF was obtained through intracranial routes in 8 studies and by lumbar puncture in 7. The most common detection method was NGS (11 studies), followed by ddPCR (5 studies). Regarding sequencing strategy, 11 studies employed targeted panels, while only 1 used a bespoke design. The most frequently analyzed biomarkers included ATRX, IDH1/2, TP53, PTEN, FUBP1, CIC, and TERT, along with alterations such as EGFR, NF1, NOTCH1, PDGFRA, CDKN2A/B, and PIK3CA. Two studies reported diagnostic accuracy in CSF, with sensitivities of 92.1% and 100%, and one reported specificity of 100%. Five studies compared ctDNA findings with MRI, all 12 reported tumor–CSF molecular concordance, 5 reported OS, 1 reported PFS, and 3 provided hazard ratios (HRs). A summary of study characteristics is presented in **Table 1**.

**Table 1:** Descriptive characteristics of included studies.

Author/year	Country	N	Study design	Study for diagnosis purposes	Study for follow-up or prognosis purposes	WHO classification 2021	Liquid biopsy Specimen type	Biomarker analyzed	Collection of CSF method	Detection method	Compared to MRI	HR reported?	HR value	Outcome (OS, PFS)	NOS score	Notes
Juratli TA et al., 2018 (11)	Germany	38	Retrospective	Yes	Yes	Grade IV	Primary tumor, CSF, Plasma	TERTp	Intracranial	NGS; ddPCR	Yes	Yes	16.02, 95% CI: 3.15–28.43	OS	8	ctDNA levels reflect tumor dynamics
Martínez-Ricarte F et al., 2018 (13)	Spain	20	Prospective	No	No	Grades II-IV	Primary tumor, CSF, Plasma	IDH1, IDH2, ATRX, TP53.	Intracranial, LP	NGS; ddPCR	No	No			8	CSF better than plasma in ctDNA detection
Li JH et al., 2019 (12)	China	5	Prospective	Yes	Yes	Grade IV	Primary tumor, CSF, Plasma	customized panel of 50 genes	LP	NGS	No	No		OS	5	CSF mutational burden better reflected sequential and post-surgical changes than plasma; no correlation with OS.
Miller AM et al., 2019 (4)	USA	85	Prospective	Yes	Yes	Grades II-IV	Primary tumor, CSF, Plasma	410 genes	Intracranial, LP	NGS	Yes	Yes	4.16 95% CI 2.15-8.05)	OS	8	ctDNA positivity in CSF was independently associated with worse OS; detection not merely a reflection of tumor volume.
Hao Duan 2020 (8)	China	9	Prospective	Yes	No	Grades II-IV	Primary tumor, CSF,Plasma	ctDNA (IDH1, H3F3A, EGFR, TP53, etc.)	LP	NGS	No	No			6	More mutations in CSF than tumor; IDH1/H3F3A concordant; WES feasible in CSF ctDNA.
Zhao Z et al., 2020 (15)	China	21	Prospective	No	Yes	Grades II-IV	Primary tumor, CSF,	68 genes	Intracranial	NGS	Yes	Yes	HR = 3.6; IC 95%: 1.2–10.8; p = 0.02.	OS	7	Positive ctDNA in CSF conferred a 3.6-fold higher risk of death. High concordance between CSF and tumor tissue; ctDNA detected mutations not seen in tissue; supports diagnostic use
Fujita Y 2022 (9)	USA	6	Prospective	Yes	Yes	Grade II	Primary tumor, CSF	50 genes	Intracranial	NGS; ddPCR	No	No			5	Combined molecular and metabolite profiling
Orzan F., 2023 (7)	Italy	84	Prospective	Yes	No	Grade II-IV	Primary tumor, CSF,Plasma	54 genes	Intracranial	ddPCR	Yes	No			6	Presence of ctDNA in CSF was associated with worse prognosis.
Wang Q., 2023 (14)	China	20	Prospective	Yes	No	Grade II-IV	Primary tumor, CSF,	IDH1/2, TP53, ATRX, EGFR, PTEN, TERTp, CIC, FUBP1, etc	LP	NGS	No	No			5	Dynamic changes in ctDNA used for monitoring
Iser F., 2024 (10)	Germany	51	Multicenter Prospective	Yes	Yes	Grade II-IV	Primary tumor, CSF,	IDH1/2, TP53, ATRX, EGFR, PTEN, TERTp, CIC, FUBP1, NF1, NOTCH1, PDGFRA, CNVs (EGFR amplification, CDKN2A/B loss, PTEN loss, etc.	Intracranial, LP	NGS	Yes	No			6	CSF ctDNA matched tumor mutations; plasma often negative
Cabezas-Camarero et al., 2025 (3)	Spain	32	Multicenter Prospective	Yes	Yes	Grade II-IV	Primary tumor, CSF, Plasma	IDH1/2, TP53, ATRX, EGFR, PTEN, PIK3CA, BRAF	Intracranial, LP	NGS	Yes	No		OS, PFS	8	HR = 3.2 (ctDNA+ in CSF associated with lower PFS). High concordance between CSF and tumor; CSF outperformed plasma; ctDNA useful for diagnosis and monitoring
Zhu Z., 2025 (16)	China	25	Multicenter Prospective	Yes	No	Grade II-IV	Primary tumor, CSF	IDH1/2, TP53, ATRX, EGFR, PTEN, TERTp, CIC, FUBP1, etc	Intracranial	NGS, ddPCR	No	No			8	Plasma ctDNA detectable in some patients

ATRX, Alpha Thalassemia/Mental Retardation Syndrome X-linked; AUC, Area Under the Curve; CI, Confidence Interval; CIC, Capicua Transcriptional Repressor; CNVs, Copy Number Variations; CSF, Cerebrospinal Fluid; ctDNA, Circulating Tumor DNA; ddPCR, Droplet Digital Polymerase Chain Reaction; EGFR, Epidermal Growth Factor Receptor; EGFRvIII, Epidermal Growth Factor Receptor variant III; FUBP1, Far Upstream Element Binding Protein 1; GBM, Glioblastoma; HR, Hazard Ratio; IDH1/2, Isocitrate Dehydrogenase 1 and 2; LP, Lumbar Puncture; MRI, Magnetic Resonance Imaging; NGS, Next Generation Sequencing; NF1, Neurofibromin 1; NOS, Newcastle–Ottawa Score;NOTCH1, Notch Homolog 1; OS, Overall Survival; PDGFRA, Platelet Derived Growth Factor Receptor Alpha; PFS, Progression Free Survival; PIK3CA, Phosphatidylinositol-4,5-Bisphosphate 3-Kinase Catalytic Subunit Alpha; PTEN, Phosphatase and Tensin Homolog; RT-PCR, Reverse Transcriptase Polymerase Chain Reaction; TERTp, Telomerase Reverse Transcriptase promoter; TP53, Tumor Protein p53; WES, Whole Exome Sequencing; WHO, World Health Organization.

**Figure 2:**
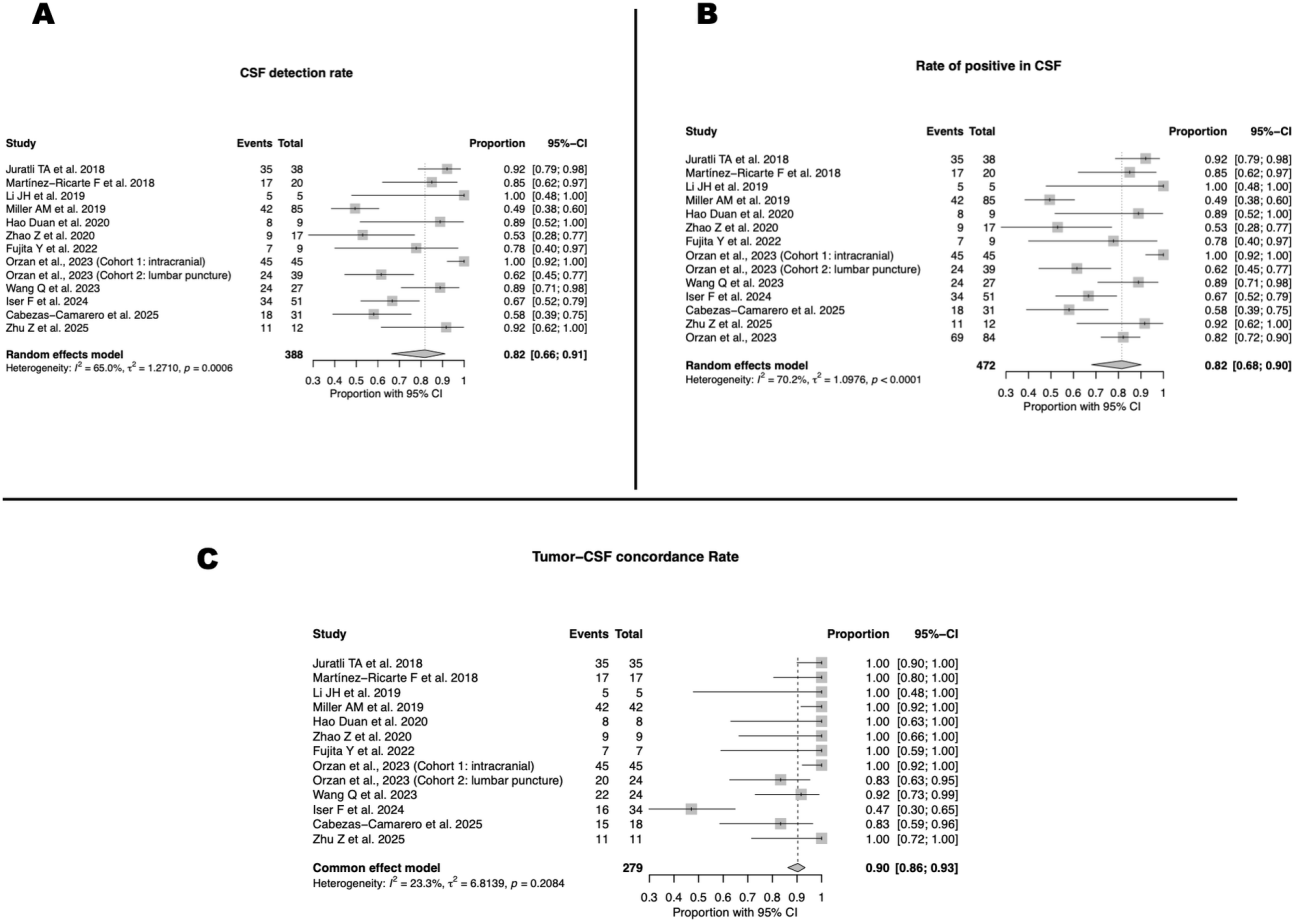
CSF ctDNA detection and tumor–CSF concordance across studies. **(A)** Pooled CSF ctDNA detection rate (82%; 95% CI, 66–91; I² = 65%). **(B)** Pooled CSF positivity rate (82%; 95% CI, 68–90; I² = 70%). **(C)** Tumor–CSF molecular concordance (90%; 95% CI, 86–93; I² = 23%).

Across the included studies, the pooled CSF detection rate was 82% (95% CI 66–91; I² = 65%), with individual series ranging from 49% to 100% and most clustering above 70% (**Figure 2A**). When considering all 472 CSF samples analyzed across the included studies, the overall CSF positivity rate remained virtually unchanged at 82% (95% CI 68–90; I²=70%), confirming the robustness of this estimate despite inter-study variability (**Figure 2B**). Molecular concordance between CSF ctDNA and matched tumor tissue was also high, with a pooled rate of 90% (95% CI 86–93; I²=23%), and while a few studies reported lower concordance values, the majority approached 100%, supporting the reliability of CSF as a faithful reflection of the tumor’s genomic profile (**Figure 2C**).

**Figure 3:**
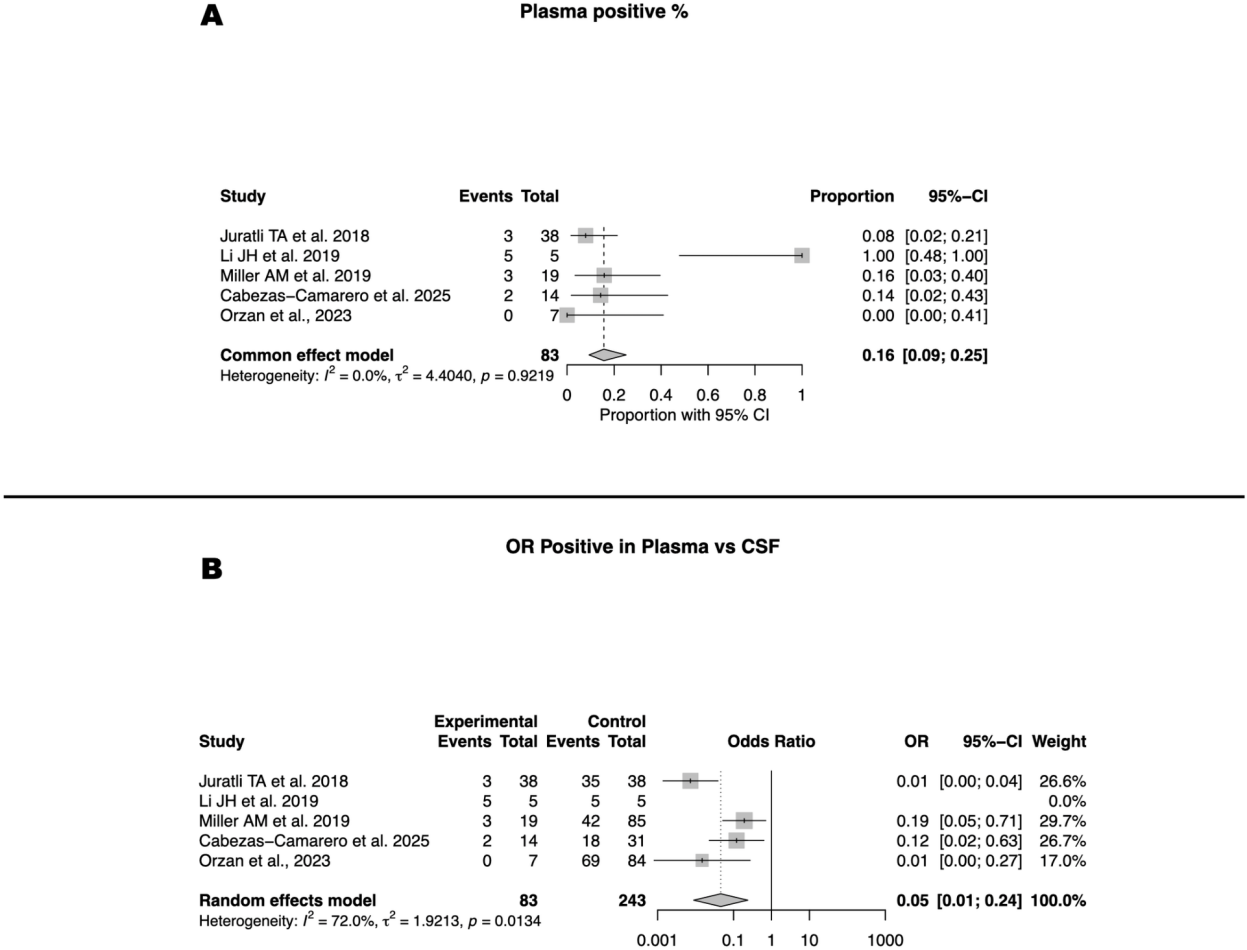
Plasma ctDNA detection and comparison with CSF. **(A)** Pooled plasma ctDNA detection rate across five studies (16%; 95% CI, 9–25; I² = 0%). **(B)** Odds ratio comparing detection in plasma versus CSF (OR = 0.05; 95% CI, 0.01–0.24; I² = 72%).

Plasma ctDNA detection was markedly limited, with a pooled positivity rate of 16% (95% CI 9–25; I² = 0%) across five studies (**Figure 3A**). Individual cohorts reported proportions ranging from 0% to 100%, but most series clustered at or below 20%, underscoring the consistently low sensitivity of plasma for glioma-derived ctDNA. When directly compared with CSF, the superiority of the latter was evident. The pooled odds ratio for plasma relative to CSF was 0.05 (95% CI 0.01–0.24; I²=72%), confirming that the likelihood of ctDNA detection in plasma is more than twenty times lower than in CSF (**Figure 3B**). Although heterogeneity was substantial, the direction of effect was consistent across studies, all of which favored CSF as the more reliable biofluid.

**Figure 4:**
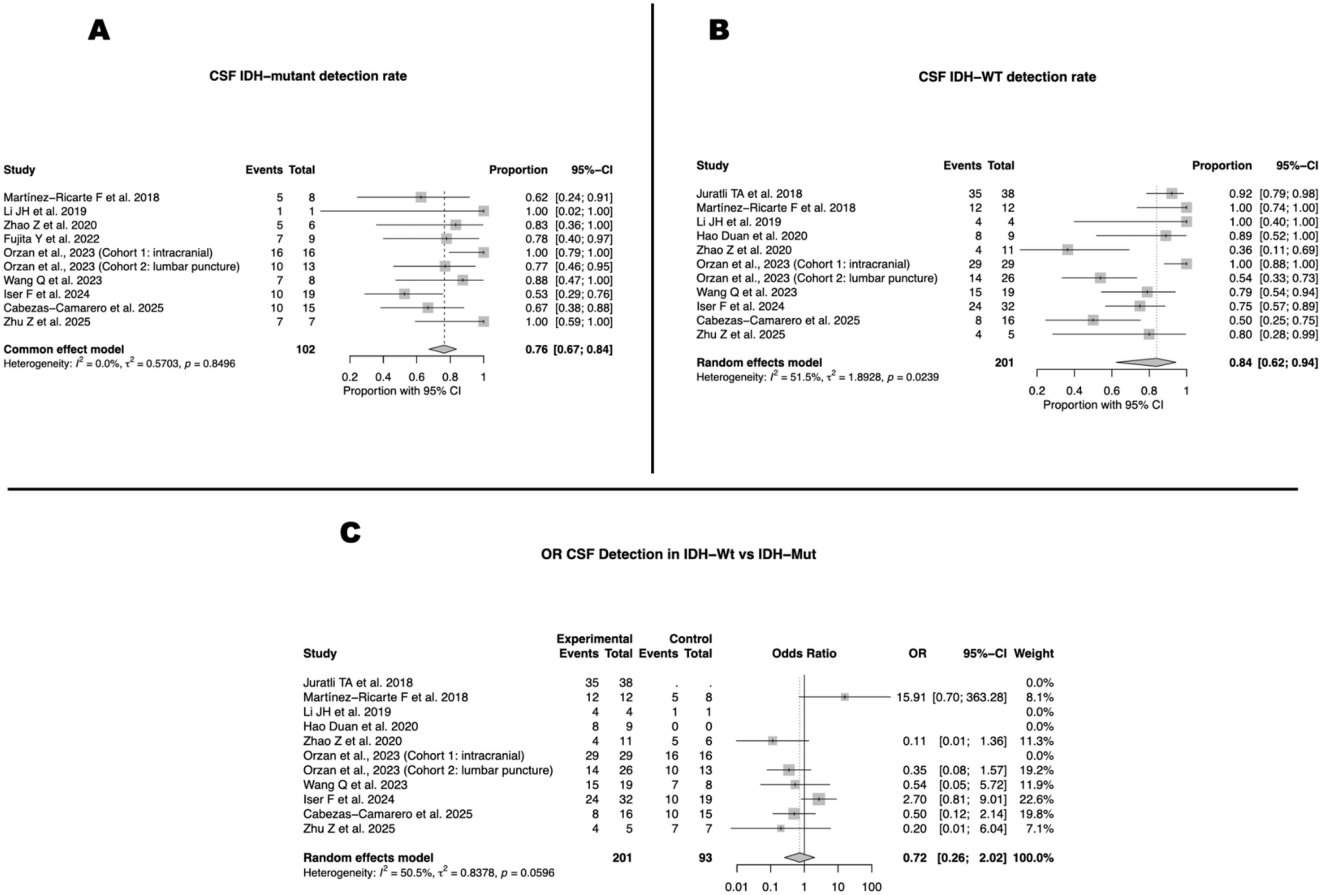
CSF ctDNA detection stratified by IDH mutational status. **(A)** Detection in IDH-mutant gliomas (76%; 95% CI, 67–84; I² = 0%). **(B)** Detection in IDH-wildtype gliomas (84%; 95% CI, 62–94; I² = 51.5%). **(C)** Odds ratio comparing IDH-wildtype versus IDH-mutant tumors (OR = 0.72; 95% CI, 0.26–2.02; I² = 50.5%).

In subgroup analyses by molecular subtype, the pooled CSF ctDNA detection rate in IDH-mutant gliomas was 76% (95% CI 67–84; I²=0%) (**Figure 4A**). By contrast, in IDH-wildtype gliomas, the detection rate was 84% (95% CI 62–94; I² =51.5%) (**Figure 4B**). Direct comparison between subgroups showed a non-significant trend toward higher detection in IDH-wildtype tumors, with a pooled odds ratio of 1.7 (95% CI 0.26–2.02; I² = 50.5%) (**Figure 4C**).

**Figure 5:**
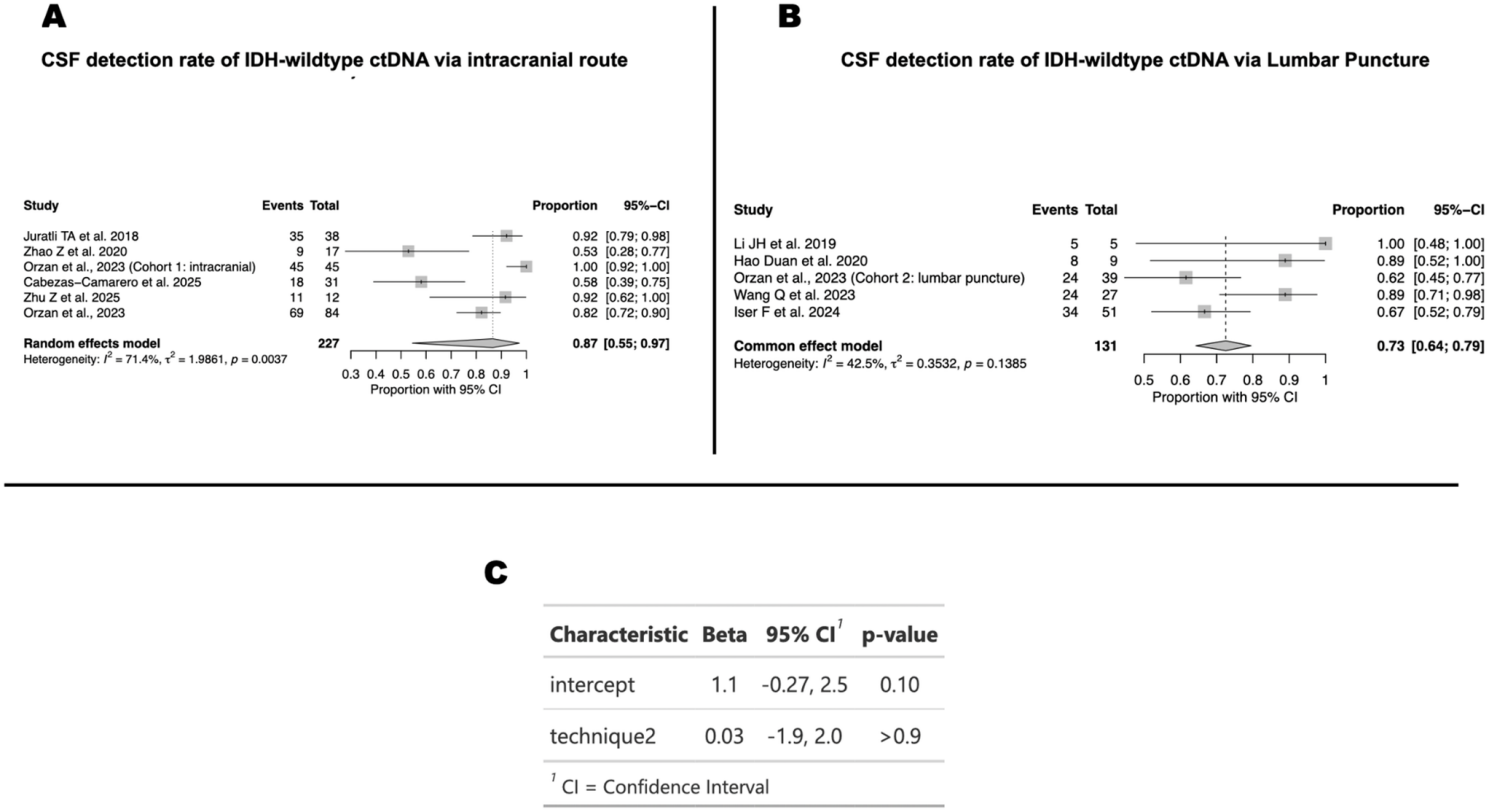
CSF collection route in IDH-wildtype gliomas. **(A)** Detection with intracranial CSF sampling (87%; 95% CI, 55–97; I² = 71.4%). **(B)** Detection with lumbar puncture (73%; 95% CI, 64–79; I² = 42.5%). **(C)** Meta-regression evaluating the association between sampling route and detection rate (β = 0.03; 95% CI, –1.9 to 2.0; p > 0.9).

In analyses restricted to IDH-wildtype gliomas, the pooled CSF detection rate was 87% (95% CI 55–97; I² = 71.4%) for intracranial sampling (**Figure 5A**) and 73% (95% CI 64–79; I² = 42.5%) for lumbar puncture (**Figure 5B**). Although point estimates suggested higher sensitivity for intracranial routes, a meta-regression including collection technique as a categorical moderator confirmed that these differences were not statistically significant (β = 0.03, 95% CI –1.9 to 2.0; p > 0.9) (**Figure 5C**).

Leave-one-out sensitivity analysis confirmed the stability of the findings. Across all proportion meta-analyses, the maximum absolute deviation of the pooled estimate relative to the primary model was ≤0.07 (≤7 percentage points), and no omission changed statistical significance. For odds ratio meta-analyses, the largest absolute change in the pooled log(OR) was ≤0.10 (approximately ≤10% relative change in OR), with no reversals in effect direction or significance. The comparison of CSF detection in IDH-wildtype versus IDH-mutant gliomas showed the largest deviation under LOO (up to 0.3 points in the estimate), but all case-deletion models remained non-significant and directionally consistent with the overall analysis.

Although survival data were limited, ctDNA positivity in CSF was consistently associated with worse outcomes across the studies.

## Discussion

This systematic review and meta-analysis synthesizes current evidence on cerebrospinal fluid (CSF)-derived circulating tumor DNA (ctDNA) as a diagnostic and prognostic biomarker in adult gliomas. Across 12 studies and 388 patients, we found that ctDNA is consistently more detectable in CSF than in plasma, with high molecular concordance to tumor tissue and emerging prognostic value. These findings highlight the promise of CSF-based liquid biopsy for diagnosis, monitoring, and prognostic stratification in glioma, while also underscoring methodological challenges that require further standardization.

Leave-one-out sensitivity analyses confirmed that no single study disproportionately influenced the pooled results, supporting the overall robustness of our conclusions across all meta-analyses. The moderate heterogeneity observed across analyses likely reflects a combination of pre-analytical/analytical and clinical/biological factors. In our dataset, variability was primarily methodological, including differences in sequencing platforms and analytic sensitivity, panel design and breadth, and definitions of ctDNA positivity, while clinical contributors such as sampling timing (diagnosis vs. recurrence or post-treatment), tumor burden and location, and histologic or molecular subtype may add secondary variability. Together, these elements can influence detection sensitivity and concordance across studies, underscoring the need for standardized pre-analytical workflows and reporting, as highlighted in prior recommendations (5, 64).

### Superiority of CSF over plasma

The most consistent result across studies is the clear superiority of CSF over plasma as a source of ctDNA in gliomas. In our pooled analysis, ctDNA detection rates in CSF reached 82%, compared with only 16% in plasma, with a pooled odds ratio of 0.05 (95% CI 0.01–0.24), confirming the markedly lower sensitivity of plasma. Tumor–CSF concordance was also high at 90% (95% CI 86–93). Although heterogeneity across studies was moderate to substantial (I² > 65%), this was largely driven by a minority of cohorts reporting lower concordance, while most series individually showed values near 100%. From a clinical perspective, this variability does not challenge the overall conclusion that CSF is the most reliable fluid for glioma-derived molecular information.

These findings align with the biological rationale that CSF directly bathes the central nervous system and provides a microenvironmental snapshot of tumor biology. Even when the blood–brain barrier is disrupted, as frequently occurs in glioblastoma, ctDNA concentrations in peripheral blood may remain below detection thresholds, whereas CSF, sampled in close anatomical proximity to the tumor, consistently contains tumor-derived nucleic acids. Prior studies (4, 65), highlighted these differences, while others (66) confirmed that plasma-based approaches remain insensitive in gliomas. Overall, the available data converge on the conclusion that CSF is the optimal biofluid for ctDNA analysis in gliomas (64). While lumbar puncture is more invasive than a blood draw, its diagnostic yield is substantially higher. The correlation between ctDNA detected in plasma and CSF is low, as plasma rarely captures the full genomic heterogeneity of gliomas due to the restrictive blood brain barrier (7, 13). Timing also influences detection, with perioperative or pretreatment CSF sampling showing the highest sensitivity and better concordance with tumor tissue.

### Factors influencing ctDNA detection in CSF

Several biological and technical factors may affect ctDNA yield in CSF. One of the most discussed is the molecular subtype, particularly IDH status. In our analysis, pooled detection rates were 76% in IDH-mutant gliomas and 84% in IDH-wildtype tumors, with a non-significant trend toward higher detection in the latter (OR 0.72, 95% CI 0.26–2.02; I² = 50.5%)The literature does not consistently demonstrate a robust difference. Orzan et al. (7) reported that ctDNA detection was feasible in both subtypes without significant differences, and other studies reached similar conclusions. Importantly, prospective work such as Fujita 2022 (9) showed that IDH1 mutations and the metabolite D-2-hydroxyglutarate can be reliably detected in CSF, but this association was limited to specific biomarkers rather than overall ctDNA levels. Similarly, Tuna et al. (67) demonstrated that IDH1 mutation status can be identified in CSF and plasma, but did not find that IDH status determined ctDNA yield. In plasma, Crucitta et al. (68) confirmed detectability and prognostic relevance of IDH1 mutations, without evidence that IDH status influences cfDNA concentration. Collectively, these results indicate that while IDH alterations can be detected, IDH status itself is not a major determinant of overall ctDNA detectability.

Another factor is the route of CSF collection. Orzan et al. (7) demonstrated higher detection when samples were obtained via ventricular routes compared with lumbar puncture, particularly with targeted panels. In contrast, our meta-analysis found no significant difference between intracranial (87%) and lumbar puncture (73%) routes, with moderate heterogeneity. Importantly, recent longitudinal studies such as the work published by Riviere-Cazaux et al. (34) have shown that intracranial access can enable repeated sampling, supporting its potential role in experimental or high-risk settings.

Sequencing strategy also plays a role. Eleven of twelve included studies used targeted panels, while only one employed a bespoke design. Evidence indicates that targeted assays, and complementary methods such as ddPCR, improve sensitivity compared with untargeted approaches. Martínez-Ricarte et al/ (13) and Guo et al., 2022 (69) confirmed their value in detecting clinically relevant alterations. Bespoke designs remain anecdotal, but targeted approaches currently provide the strongest evidence for clinical translation.

### Molecular concordance

A key question is whether ctDNA faithfully reflects tumor genomics. In our analysis, concordance between CSF ctDNA and matched tumor tissue was 90% (95% CI 86–93) Most cohorts reported values near 100%, while a few contributed lower estimates (as low as 48%). These outliers explain statistical heterogeneity but do not undermine the conclusion that CSF reliably mirrors the glioma mutational landscape. Moreover, several reports identified mutations in CSF not found in tumor tissue, suggesting that liquid biopsy can capture intratumoral heterogeneity and subclonal dynamics missed in surgical specimens (7, 13, 69) These findings are aligned with current evidence on key molecular biomarkers in glioblastoma, including MGMT promoter methylation, IDH1/2 mutations, EGFR amplification, and TERT promoter mutations, which define distinct prognostic and therapeutic subgroups (70). Thus, CSF ctDNA is not simply a surrogate of tissue testing but a complementary tool that may provide broader genomic insight.

### Prognostic value

Whether ctDNA carries prognostic implications is a critical question. As summarized in **Table 1**, only five of the twelve included studies reported overall survival and one reported progression-free survival data. Given this limited and heterogeneous reporting, a pooled meta-analysis of prognostic outcomes was not feasible. Nevertheless, across all studies that assessed survival, ctDNA positivity in CSF was consistently associated with shorter OS and/or PFS, supporting its potential prognostic value despite the scarcity of quantitative data. In our previous prospective multicenter study (3) ctDNA positivity in CSF was consistently associated with worse outcomes. Patients with positive ctDNA and a variant allele fraction (VAF) at or above the median had significantly shorter progression-free survival (HR 3.2) compared with those below the median, and both PFS and OS were reduced in ctDNA-positive patients.

Other studies provide supporting evidence. Hickman et al (71), in a clinical cohort of patients with CNS tumors, found ctDNA positivity correlated with poor outcomes, while Juratli et al. (11) linked promoter mutations in CSF to aggressive glioblastoma. Collectively, these findings reinforce the potential of CSF ctDNA as a prognostic biomarker, although larger prospective cohorts with harmonized endpoints are needed for validation.

### Clinical timing, tumor characteristics, and integration with other biomarkers

Most studies collected CSF perioperatively, and evidence on longitudinal monitoring remains limited. Sampling time varied across studies, most commonly performed perioperatively or at recurrence. Some reports described ctDNA dynamics in CSF that paralleled or anticipated radiographic changes, supporting its potential role for early progression detection (13, 34, 67). Recent data demonstrate feasibility (34**).** et al. observed dynamic ctDNA fluctuations with treatment and progression, often preceding MRI changes, even in pseudo-progression contexts. The correlation between ctDNA and imaging is encouraging but imperfect. Declines after surgery or chemoradiotherapy typically paralleled tumor shrinkage on MRI, while rises often preceded radiological progression. However, mismatches occur, and optimal thresholds and timing remain undefined. Still, ctDNA can reveal molecular alterations when imaging is equivocal, aiding the distinction between true progression and treatment effects (3, 16).

Regarding tumor characteristics, our previous prospective study (3) found no significant association between ctDNA detectability in CSF and tumor size or distance to ventricular reservoirs. Earlier studies such as Orzan et al. (7) and Martínez-Ricarte et al. (13) had suggested these variables might influence shedding, but current evidence indicates they remain unproven hypotheses. As such, they should be considered biologically plausible but not validated determinants, pending larger prospective confirmation (14). Recent studies have highlighted the relevance of ligand-gated ion channels (LGICs) in glioma biology. Alterations in purinergic, glutamatergic, and Cys-loop receptor families have been linked to tumor progression and neurological dysfunction, supporting their potential as biomarkers and therapeutic targets (72). Although these mechanisms fall outside the scope of ctDNA analysis, integrating molecular and electrophysiological biomarkers could further refine glioma characterization.

Plasma ctDNA and circulating tumor cells, by contrast, continue to show poor performance, reinforcing CSF as the biofluid of choice for glioma molecular profiling (3, 71). From a procedural standpoint, CSF collection is not risk-free. Lumbar puncture is generally safe when mass effect or obstructive hydrocephalus are excluded, with post-puncture headache occurring in up to 11% (4.2% with atraumatic needles) and serious complications such as infection (<0.1%) or herniation (<1%) being rare (73). Intracranial reservoirs allow repeated sampling but carry infection rates of 2–10% and occasional mechanical or hemorrhagic complications (74). These risks should be balanced against the potential diagnostic benefit in each case. Regional differences in access to molecular testing, sequencing platforms, and feasibility of CSF sampling may influence how liquid biopsy is implemented across centers, underscoring the need for harmonized, evidence-based algorithms to guide clinical decision-making.

Finally, the integration of CSF ctDNA with advanced imaging and other biomarkers represents a promising frontier. Combined approaches may improve sensitivity and specificity for progression detection and therapeutic monitoring. Recent work illustrates this shift: Dwarshuis el al (75). highlighted the utility of liquid biopsy alongside imaging in gliomas and metastases and Zheng et al. (76)demonstrated that CSF ctDNA could stratify prognosis and guide therapy in CNS metastases. This body of evidence supports the role of CSF ctDNA within a broader diagnostic ecosystem rather than as an isolated tool.

## Limitations

This study has limitations. The modest number of included studies and patients restricts generalizability. Substantial heterogeneity in collection techniques, detection platforms, and endpoints precluded pooled analyses for some outcomes, particularly survival and longitudinal monitoring. Risk of bias was low to moderate, but common limitations included small sample size, variable follow-up, and incomplete adjustment for confounders. Differences in mutational panels and reporting thresholds further complicate comparisons. Standardized methodologies are urgently needed.

## Conclusions and future perspectives

This systematic review and meta-analysis confirms that CSF is the most informative biofluid for ctDNA detection in gliomas, with higher sensitivity than plasma and strong concordance with tumor tissue. The effect of IDH status appears weaker than previously suggested, and CSF collection route did not significantly influence detection in pooled analyses. Importantly, ctDNA positivity is associated with worse prognosis, underscoring its potential as a biomarker for prognostic stratification.

Overall, while factors such as IDH status, CSF collection route, and sequencing platform may influence detection rates, none consistently determines ctDNA positivity across studies. This emphasizes the need for large-scale prospective investigations to identify robust predictors and standardize methodologies for clinical translation.

Future research should prioritize the standardization of CSF sampling and analytical methods, as highlighted by the RANO group (5), to ensure reproducibility and clinical applicability. Multicenter prospective studies are required to validate the prognostic and predictive role of CSF ctDNA, and emerging ultra-sensitive sequencing and point-of-care technologies may enable real-time molecular monitoring and integration with imaging and clinical data for precision-guided management of gliomas.

## Data Availability

The original contributions presented in the study are included in the article/**Supplementary Material**. Further inquiries can be directed to the corresponding author.
