# Supplementary material for: Cerebrospinal fluid ctDNA as a diagnostic and prognostic tool in gliomas: a systematic review and meta-analysis
Source: Front Oncol. 2025 Dec 11;15:1714287. doi: 10.3389/fonc.2025.1714287 (PMC12738317; doi:10.3389/fonc.2025.1714287)
Supplement: Supplementary file 1 [file DataSheet1.docx]

# Supplementary Material 1: Studies included and excluded according to the eligibility criteria, following the PRISMA workflow

| **Supplementary Table 1. Articles included in the analysis**   \| **Author / Year** \| **Title** \| \| --- \| --- \| \| Cabezas-Camarero et al. 2025^3^ \| ctDNA detection in cerebrospinal fluid and plasma and mutational concordance with the primary tumor in a multicenter prospective study of patients with glioma \| \| Hao Duan 2020^8^ \| Assessment of circulating tumor DNA in cerebrospinal fluid by whole exome sequencing to detect genomic alterations of glioblastoma \| \| Yoko Fujita 2022^9^ \| IDH1 p.R132H ctDNA and D-2-hydroxyglutarate as CSF biomarkers in patients with IDH-mutant gliomas \| \| Iser F et al 2024^10^ \| Cerebrospinal Fluid cfDNA Sequencing for Classification of Central Nervous System Glioma \| \| Juratli TA et al 2018^11^ \| TERT Promoter Mutation Detection in Cell-Free Tumor-Derived DNA in Patients with IDH Wild-Type Glioblastomas: A Pilot Prospective Study \| \| Li JH et al 2019^12^ \| Assessment of ctDNA in CSF may be a more rapid means of assessing surgical outcomes than plasma ctDNA in glioblastoma \| | \| **Author / Year** \| **Title** \| \| --- \| --- \| \| Martínez-Ricarte F et al 2018^13^ \| Molecular Diagnosis of Diffuse Gliomas through Sequencing of Cell-Free Circulating Tumor DNA from Cerebrospinal Fluid \| \| Miller AM et al 2019^4^ \| Tracking tumour evolution in glioma through liquid biopsies of cerebrospinal fluid \| \| Orzan F et al 2023^7^ \| Liquid Biopsy of Cerebrospinal Fluid Enables Selective Profiling of Glioma Molecular Subtypes at First Clinical Presentation \| \| Wang Q et al, 2023 ^14^ \| Concordance analysis of cerebrospinal fluid with the tumor tissue for integrated diagnosis in gliomas based on next-generation sequencing \| \| Zhao Z et al 2020^15^ \| Applications of cerebrospinal fluid circulating tumor DNA in the diagnosis of gliomas \| \| Zhu Z et al, 2025^16^ \| Cerebrospinal Fluid-Derived Genomic Alterations Tracking Glioma \| |
| --- | --- | --- | --- | --- | --- | --- | --- | --- | --- | --- | --- | --- | --- | --- | --- | --- | --- | --- | --- | --- | --- | --- | --- | --- | --- | --- | --- | --- | --- |

| **Supplementary Table 2. Articles excluded from the analysis**   \| **Author** \| **Reason for rejection** \| \| --- \| --- \| \| Ali Abasi et al^17^ \| Pediatric population \| \| Tej D Azad et al^18^ \| Review, pediatric population \| \| S. Bagley et al^73^ \| CfDNA \| \| L. balager-Lluna et al^19^ \| Pediatric population \| \| L.Y Ballester et al^43^ \| Include other type of tumors (metastasis) \| \| Batool SM et al^74^ \| Extracelular vesicle RNA \| \| M.T. Bounajem et al^49^ \| Review, pediatric population \| \| Buzova D et al^20^ \| Pediatric population \| \| Cabezas-Camarero et al^75^ \| Subsets of published cohorts \| \| Canton E et al ^21^ \| Pediatric population \| \| Chahardehi AM et al^50^ \| Review \| \| Chai R el al. 2024^45^ \| Spinal cord gliomas \| \| Di WY et al^46^ \| Spinal cord astrocitomas \| \| Eibl RH et al ^51^ \| Review \| \| Gavrjushin AV et al^22^ \| Pediatric population \| \| Gavrjushin AV et al,^76^ \|  \| \| George SL et al^23^ \| Pediatric population \| \| Greuter L et al^24^ \| Pediatric population \| \| Husain A et al^36^ \| cfDNA \| \| Iorgulescu JB et al^33^ \| cfDNA \| \| Jovčevska I et al^52^ \| Review \| \| Kolostova K et al. ^39^ \| Circulating cell tumor (CTC) and cfDNA \| \| Krynina O et al ^25^ \| Pediatric population \| \| Śledzińska P et al ^53^ \| Review \| \| Lennartz P et al^41^ \| Vesicular and circulating Hsp70 \| \| Le Rhun E et al^54^ \| Review \| \| Liang J et al^44^ \| Include other type of tumors (metastasis) \| \| Li D et al^26^ \| Pediatric population \|   **Supplementary Table 3. Newcastle–Ottawa Scale (NOS) quality assessment of included studies** | \| **Author** \| **Reason for rejection** \| \| --- \| --- \| \| Liu G et al 2023^77^ \| No CSF analysis \| \| Lu VM et al^55^ \| Review \| \| Lu Y et al^56^ \| Review \| \| Madlener S et al^6^ \| Pediatric population \| \| McMahon JT et al^64^ \| Metaanalysis \| \| Meng Y et al^78^ \| No CSF analysis \| \| Nakano Y et al^47^ \| Case report \| \| Natsumeda M et al^57^ \| Review \| \| On J et al^27^ \| Pediatric population \| \| Otsuji R et al^2^ \| cfDNA \| \| Panditharatna E et al^79^ \| Pediatric population \| \| Patel J et al ^58^ \| Review \| \| Penkova A et al^59^ \| Review \| \| Pieri V et al^48^ \| Case Report \| \| Riviere-Cazaux C et al^34^ \| cfDNA \| \| Ronsley R et al ^80^ \| Pediatric population \| \| Salviano-Silva A et al ^42^ \| Extracellular vesicles \| \| Seyhan AA ^60^ \| Review \| \| Shibuma S et al ^28^ \| Pediatric population, known leptomeningeal disease \| \| Simonelli M et al ^61^ \| Review \| \| Stankunaite R et al ^29^ \| Pediatric population \| \| Tuna G et al ^35^ \| cfDNA \| \| Vaidya M et al^62^ \| Review \| \| Wesseling P et al ^63^ \| Review \| \| Zhang S et al ^36^ \| cfDNA \| \| Mouliere F et al ^37^ \| cfDNA \| \| Szadkowska P et al ^38^ \| ccfDNA \| \| Pan C et al 2019^30^ \| Pediatric patients \| |
| --- | --- | --- | --- | --- | --- | --- | --- | --- | --- | --- | --- | --- | --- | --- | --- | --- | --- | --- | --- | --- | --- | --- | --- | --- | --- | --- | --- | --- | --- | --- | --- | --- | --- | --- | --- | --- | --- | --- | --- | --- | --- | --- | --- | --- | --- | --- | --- | --- | --- | --- | --- | --- | --- | --- | --- | --- | --- | --- | --- | --- | --- | --- | --- | --- | --- | --- | --- | --- | --- | --- | --- | --- | --- | --- | --- | --- | --- | --- | --- | --- | --- | --- | --- | --- | --- | --- | --- | --- | --- | --- | --- | --- | --- | --- | --- | --- | --- | --- | --- | --- | --- | --- | --- | --- | --- | --- | --- | --- | --- | --- | --- | --- | --- | --- | --- | --- | --- |

| **Author / Year** | **NOS Score** | **NOS quality** | **Risk of Bias** |
| --- | --- | --- | --- |
| Juratli TA et al 2018^11^ | 8 | 8 (High) | Low |
| Martínez-Ricarte F et al 2018^13^ | 8 | 8 (High) | Low |
| Li JH et al 2019^12^ | 5 | 5 (Moderate) | Moderate |
| Miller AM et al 2019^4^ | 8 | 8 (High) | Low |
| Hao Duan 2020^8^ | 6 | 6 (Moderate) | Moderate |
| Zhao Z et al 2020^15^ | 7 | 7 (High) | Low |
| Fujita Y 2022^9^ | 5 | 5 (Moderate) | Moderate |
| Orzan F., 2023^7^ | 6 | 6 (Moderate) | Moderate |
| Wang Q., 2023^14^ | 5 | 5 (Moderate) | Moderate |
| Iser F., 2024^10^ | 6 | 6 (Moderate) | Moderate |
| Cabezas-Camarero et al., 2025^3^ | 8 | 8 (High) | Low |
| Zhu Z., 2025^16^ | 8 | 8 (High) | Low |

The Newcastle–Ottawa Scale (NOS) evaluates observational studies across three domains (selection, comparability, and outcome) with a maximum score of 9. Studies were classified as low risk (score ≥7), moderate risk (score 5–6), or high risk (score <5).
